# Supplementary material for: Bromodomain Factor 5 as a Target for Antileishmanial Drug Discovery
Source: ACS Infect Dis. 2023 Oct 31;9(11):2340–57. doi: 10.1021/acsinfecdis.3c00431 (PMC10644352; doi:10.1021/acsinfecdis.3c00431)
Supplement: Supplementary file 1 — id3c00431_si_001.pdf [file id3c00431_si_001.pdf]

Bromodomain Factor 5 as a Target for Antileishmanial Drug Discovery – Supporting Information File

Catherine N. Russell[1], Jennifer L. Carter[2], Juliet M. Borgia[1], Jacob Bush[3], Félix Calderón[4], Raquel Gabarró[4], Stuart J. Conway[2], Jeremy C. Mottram[5], Anthony J. Wilkinson[1], Nathaniel G. Jones[5] *

Affiliations:

[1]York Structural Biology Laboratory and York Biomedical Research Institute, Department of Chemistry, University of York, York, YO10 5DD, UK
[2]Department of Chemistry, Chemistry Research Laboratory, University of Oxford, Mansfield Road, Oxford, OX1 3TA, UK
[3]GSK, Gunnels Wood Road, Stevenage, Hertfordshire, SG1 2NY, UK
[4]GSK Global Health, Tres Cantos, 28760 Madrid, Spain
[5]York Biomedical Research Institute, Department of Biology, University of York, York, YO10 5NG, UK
* Corresponding author; email: nathaniel.jones@york.ac.uk

Table S1. *Ld*BDF5 BD5.1 recombinant protein details; DNA sequence codon-optimised for *E. coli*.

**His$_6$-*Ld*BDF5 BD5.1**	
Plasmid	pGL2776 pET-15-LdBDF5.1
LDBPK_091320 amino acids	5-122
DNA Sequence	ATGCATCATCATCATCATCACAGCAGCGGCAGAGAAAACTTGTATTTCCAGGGCC<br>CGAAGCTCTATAATGAGGCAGATGTAGCTGCGCTGGTGCGGAGCCTGGATCGTGC<br>GGAGGATCATCATATCTTTGCCGTCGATGTTCTGGAAACCTATCCGTACTTAGCGG<br>AAAGCTACACCAAAGTATGCCCGCGTCGCTGCGATTTGGCGACCGCAGCCCAAAA<br>GGCACTGGAGGGAGCTTATAGCTACGACCTGCGGTTGGAAGGCCTCAAAGCCGAC<br>ATCGCTCTTATGGCGTCTAACTGCGTCGCCTATAATGGGCCGACGAGCGCGTATGC<br>CGAAACTGCCGCCAAATTTGAACGGTATGCCCTGGAACAGATTGATGCTTTCGTGT<br>TGGAACATAACGGGGGTTGCTGATGA
Amino Acid Sequence	MHHHHHHSSGRENLYFQGPKLYNEADVAALVRSLDRAEDHHIFAVDVLETYPYLAES<br>YTKVCPRRCDLATAAQKALEGAYSYDLRLEGLKADIALMASNCVAYNGPTSAYAET<br>AAKFERYALEQIDAFVLEHNGGC
Number of Amino Acids	136
Molecular Mass (Da)	15191
ε (M^{-1} cm^{-1})	15150

Table S2. *Ld*BDF5 BD5.2 recombinant protein details; DNA sequence codon-optimised for *E. coli*.

**His$_6$-*Ld*BDF5 BD5.2**	
Plasmid	pGL2775 pET-15-LdBDF5.2
LDBPK_091320 amino acids	162 - 300
DNA Sequence	ATGCATCATCATCATCATCACAGCAGCGGCAGAGAAAACTTGTATTTCCAGGGCG<br>CGGCGCCTCCAAGTACACGTGAGATGGTTCAGCTGGTTGATAGCCTTAATCGTCGC<br>GAAGATGGTGGCGCCTTTTCCGTTGATGTAGCAGAAGCATACCCCGATTTGCGCGA<br>CAGTTATCGCAAAATTTGTCCACGCCCTATGAACCTGATTCTGATGCGCCAACGCG<br>CTAAAGAGGGTTACTACACCTCGGGGTCTGCCACGGTTTACGGTGACACGGTAGC<br>AGCATCCCTGACGCGCTTGCGTGAGGACATTGAGCTGTTAGTGCGCAACTGTATCA<br>CATTCAATGTCAAAGTAGAGTCTTGGGTCACTTTGGCACGCAGCTTCCAAGCGTTC<br>GCGCACCGTCGCGTGGACGACTTCGTGCTTCGCCACGCAGCATTCCTGCGTGGGAC<br>GACCATGGGCGCGGAAGTTTACGAGTGATGA
Amino Acid Sequence	MHHHHHHSSGRENLYFQGAAPPSTREMVQLVDSLNRREDGGAFSVDVAEAYPDLRD<br>SYRKICPRPMNLILMRQRAKEGYYTSGSATVYGDTVAASLTRLREDIELLVRNCITFNV<br>KVESWVTLARSFQAFAHRRVDDFVLRHAAFLRGTTMGAEVYE
Number of Amino Acids	157
Molecular Mass (Da)	17931
ε (M^{-1} cm^{-1})	16055

Table S3. His-tagged and His-tag cleaved *Ld*BDF5 BD5T recombinant protein details; DNA sequence codon-optimised for *E. coli*.

**His$_6$-*Ld*BDF5 BD5T**	
Plasmid	pLdBDF5BD5T
LDBPK_091320 amino acids	1-300
DNA Sequence (His-tagged)	ATGCATCATCATCATCATCACAGCAGCGGCAGAGAAAACTTGTATTTCCAGGGCA<br>TGTCGGCCACCCCGAAGCTCTATAATGAGGCAGATGTAGCTGCGCTGGTGCGGAG<br>CCTGGATCGTGCGGAGGATCATCATATCTTTGCCGTCGATGTTCTGGAAACCTATC<br>CGTACTTAGCGGAAAGCTACACCAAAGTATGCCCGCGTCGCTGCGATTTGGCGAC<br>CGCAGCCCAAAAGGCACTGGAGGGAGCTTATAGCTACGACCTGCGGTTGGAAGGC<br>CTCAAAGCCGACATCGCTCTTATGGCGTCTAACTGCGTCGCCTATAATGGGCCGAC<br>GAGCGCGTATGCCGAAACTGCCGCCAAATTTGAACGGTATGCCCTGGAACAGATT<br>GATGCTTTCGTGTTGGAACATAACGGGGGTTGCCGTGTAAGCCGTTTGCGTTTGCC<br>CCGGGCCTCGGCTAGTCAGGAACATGCTAGTGCAGACGGTACTGCTCCAAAGAAG<br>GGGTCCGCCGGGACCTCAGCTGCGCATAAAACTGCTGCGGCGGCGCCTCCAAGTA<br>CACGTGAGATGGTTCAGCTGGTTGATAGCCTTAATCGTCGCGAAGATGGTGGCGC<br>CTTTTCCGTTGATGTAGCAGAAGCATACCCCGATTTGCGCGACAGTTATCGCAAAA<br>TTTGTCCACGCCCTATGAACCTGATTCTGATGCGCCAACGCGCTAAAGAGGGTTAC<br>TACACCTCGGGGTCTGCCACGGTTTACGGTGACACGGTAGCAGCATCCCTGACGC<br>GCTTGCGTGAGGACATTGAGCTGTTAGTGCGCAACTGTATCACATTCAATGTCAAA<br>GTAGAGTCTTGGGTCACTTTGGCACGCAGCTTCCAAGCGTTCGCGCACCGTCGCGT<br>GGACGACTTCGTGCTTCGCCACGCAGCATTCCTGCGTGGGACGACCATGGGCGCG<br>GAAGTTTACGAGTGATGA
Amino Acid Sequence (His-tagged)	MHHHHHHSSGRENLYFQGMSATPKLYNEADVAALVRSLDRAEDHHIFAVDVLETYP<br>YLAESYTKVCPRRCDLATAAQKALEGAYSYDLRLEGLKADIALMASNCVAYNGPTSA<br>YAETAAKFERYALEQIDAFVLEHNGGCRVSRLRLPRASASQEHASADGTAPKKGSAG<br>TSAAHKTAAAAPPSTREMVQLVDSLNRREDGGAFSVDVAEAYPDLRDSYRKICPRPM<br>NLILMRQRAKEGYYTSGSATVYGDTVAASLTRLREDIELLVRNCITFNVKVESWVTLA<br>RSFQAFAHRRVDDFVLRHAAFLRGTTMGAEVYE
Number of Amino Acids	318
Molecular Mass (His-tagged) (Da)	35185
ε (M^{-1} cm^{-1})	29715
***Ld*BDF5 BD5T**	
DNA Sequence (His-tag cleaved)	GGCATGTCGGCCACCCCGAAGCTCTATAATGAGGCAGATGTAGCTGCGCTGGTGC<br>GGAGCCTGGATCGTGCGGAGGATCATCATATCTTTGCCGTCGATGTTCTGGAAACC<br>TATCCGTACTTAGCGGAAAGCTACACCAAAGTATGCCCGCGTCGCTGCGATTTGGC<br>GACCGCAGCCCAAAAGGCACTGGAGGGAGCTTATAGCTACGACCTGCGGTTGGAA<br>GGCCTCAAAGCCGACATCGCTCTTATGGCGTCTAACTGCGTCGCCTATAATGGGCC<br>GACGAGCGCGTATGCCGAAACTGCCGCCAAATTTGAACGGTATGCCCTGGAACAG<br>ATTGATGCTTTCGTGTTGGAACATAACGGGGGTTGCCGTGTAAGCCGTTTGCGTTT<br>GCCCCGGGCCTCGGCTAGTCAGGAACATGCTAGTGCAGACGGTACTGCTCCAAAG<br>AAGGGGTCCGCCGGGACCTCAGCTGCGCATAAAACTGCTGCGGCGGCGCCTCCAA<br>GTACACGTGAGATGGTTCAGCTGGTTGATAGCCTTAATCGTCGCGAAGATGGTGG<br>CGCCTTTTCCGTTGATGTAGCAGAAGCATACCCCGATTTGCGCGACAGTTATCGCA<br>AAATTTGTCCACGCCCTATGAACCTGATTCTGATGCGCCAACGCGCTAAAGAGGGT<br>TACTACACCTCGGGGTCTGCCACGGTTTACGGTGACACGGTAGCAGCATCCCTGAC<br>GCGCTTGCGTGAGGACATTGAGCTGTTAGTGCGCAACTGTATCACATTCAATGTCA<br>AAGTAGAGTCTTGGGTCACTTTGGCACGCAGCTTCCAAGCGTTCGCGCACCGTCGC<br>GTGGACGACTTCGTGCTTCGCCACGCAGCATTCCTGCGTGGGACGACCATGGGCG<br>CGGAAGTTTACGAGTGATGA
Amino Acid Sequence (His-tag cleaved)	GMSATPKLYNEADVAALVRSLDRAEDHHIFAVDVLETYPYLAESYTKVCPRRCDLAT<br>AAQKALEGAYSYDLRLEGLKADIALMASNCVAYNGPTSAYAETAAKFERYALEQIDA<br>FVLEHNGGCRVSRLRLPRASASQEHASADGTAPKKGSAGTSAAHKTAAAAPPSTREM

	VQLVDSLNRREDGGAFSVDVAEAYPDLRDSYRKICPRPMNLILMRQRAKEGYYTSGS ATVYGDTVAASLTRLREDIELLVRNCITFNVKVESWVTLARSFQAFAHRRVDDFVLRH AAFLRGTTMGAEVYE
Number of Amino Acids	301
Molecular Mass (His-tag cleaved) (Da)	33048
ε (M^{-1} cm^{-1})	28225

Table S4. His-tagged and His-tag cleaved *Ld*BDF2 recombinant protein details.

**His_6-*Ld*BDF2 BD**	
Plasmid	pGL2772 pET-15-LdBDF2
LDBPK_363130 amino acids	1-125
DNA Sequence	CATCATCATCATCATCACAGCAGCGGCAGAGAAAACTTGTATTTCCAGGGCATGG ACGTCAGCAAGCGGCCACGCGAGGAATTCCACAAGGAGCAGTGTCTCTCCTTCGT GAAGAAGCTTTGGGCGGCCGACACGCTCGCCATGTTTCACTATCCGGTGAGCGCC ACCGAGGTGCCCGGCTACTACGACGTCGTAGATACACCTATGGACCTGTCCACGA TTCGGAAGAACATCGAGCAGGGCAAGTACAGAACGGACACTGAGGTCGAGAATG ACGTGGTGCTCATGCTGTCGAACGCTCTGGACTTTAACGAGAAAGGCTCGCAATG GCATGATTTGGCGAAGCAGCTCAAGAAGCGGTACCTGACCCTTGCGCAGGAGTCG GGGCTGTCCTTCGACGCAGACCAGGCGTTCATCCCTACGAAATGATGA
Amino Acid Sequence	MGSSHHHHHHSSGLEVLFQGPAMDVSKRPREEFHKEQCLSFVKKLWAADTLAMFHY PVSATEVPGYYDVVDTPMDLSTIRKNIEQGKYRTDTEVENDVVLMLSNALDFNEKGS QWHDLAKQLKKRYLTLAQESGLSFDADQAFIPTK
Number of Amino Acids	143
Molecular Mass (Da)	16615
ε (M^{-1} cm^{-1})	19940
***Ld*BDF2 BD**	
DNA Sequence	GGCATGGACGTCAGCAAGCGGCCACGCGAGGAATTCCACAAGGAGCAGTGTCTCT CCTTCGTGAAGAAGCTTTGGGCGGCCGACACGCTCGCCATGTTTCACTATCCGGTG AGCGCCACCGAGGTGCCCGGCTACTACGACGTCGTAGATACACCTATGGACCTGT CCACGATTCGGAAGAACATCGAGCAGGGCAAGTACAGAACGGACACTGAGGTCG AGAATGACGTGGTGCTCATGCTGTCGAACGCTCTGGACTTTAACGAGAAAGGCTC GCAATGGCATGATTTGGCGAAGCAGCTCAAGAAGCGGTACCTGACCCTTGCGCAG GAGTCGGGGCTGTCCTTCGACGCAGACCAGGCGTTCATCCCTACGAAATGATGA
Amino Acid Sequence	GMDVSKRPREEFHKEQCLSFVKKLWAADTLAMFHYPVSATEVPGYYDVVDTPMDLS TIRKNIEQGKYRTDTEVENDVVLMLSNALDFNEKGSQWHDLAKQLKKRYLTLAQESG LSFDADQAFIPTK
Number of Amino Acids	126
Molecular Mass (Da)	14478
ε (M^{-1} cm^{-1})	18450

Table S5. *Ld*BDF5 BD5.2 apo structure crystallographic data collection and refinement statistics. [a] Values in parentheses correspond to the outer resolution shell. $R_{merge} = \sum_{hkl}\sum_i|I_i - \langle I\rangle| / \sum_{hkl}\sum_i \langle I\rangle$ where I_i is the intensity of the *i*th measurement of a reflection with indexes *hkl* and $\langle I\rangle$ is the statistically weighted average reflection intensity. [d] $Rpim = \sum_{hkl}[1/(n-1)]^{1/2}\sum_i|I_i - \langle I\rangle|/\sum_{hkl}\sum_i I$ Rwork $= \sum||F_o| - |F_c||/\sum|F_o|$ where F_o and F_c are the observed and calculated structure factor amplitudes, respectively. *R*free is the *R*-factor calculated with 5% of the reflections chosen at random and omitted from refinement. Root-mean-square deviation of bond lengths and bond angles from ideal geometry. Percentage of residues in most-favoured/additionally allowed/generously allowed/disallowed regions of the Ramachandran plot, according to PROCHECK.

	*Ld*BDF5 BD5.2 PDB ID: **8BPT**
**Data collection**	
Beamline/Date	i04 01/07/2018
Wavelength (Å)	0.979500Å
Space Group	$P2_12_12_1$
Cell dimensions	
a, *b*, *c* (Å)	33.4 75.4 105.8
α, β, γ (°)	90.0, 90.0, 90.0
Resolution (Å) [a]	31.82 - 1.60 (1.63-1.60)
R_{merge} [a,b]	0.049 (0.600)
Rmeas [a,c]	0.053 (0.639)
Rpim [a,d]	0.018 (0.217)
CC(1/2)	0.999 (0.928)
$I / (\sigma I)$ [a]	19.5 (2.9)
Completeness (%) [a]	98.3 (97.1)
Observations [a]	285,068 (14,599)
Unique Reflections [a]	35,399 (1,738)
Wilson B estimate ($Å^2$)	19.20
**Refinement**	
Resolution (Å)	31.82-1.60
No. reflections	35,359 (1,735)
R_{work} / R_{free}	0.171 / 0.230
No. atoms	
Protein	2212
Water	147
B-factors	
Protein	18.01
Water	32.98
R.m.s. deviations	
Bond lengths (Å)	0.0138
Bond angles (°)	1.874
Ramachandran Statistics (%)	
Preferred	98.91
Allowed	1.09
Outliers	0

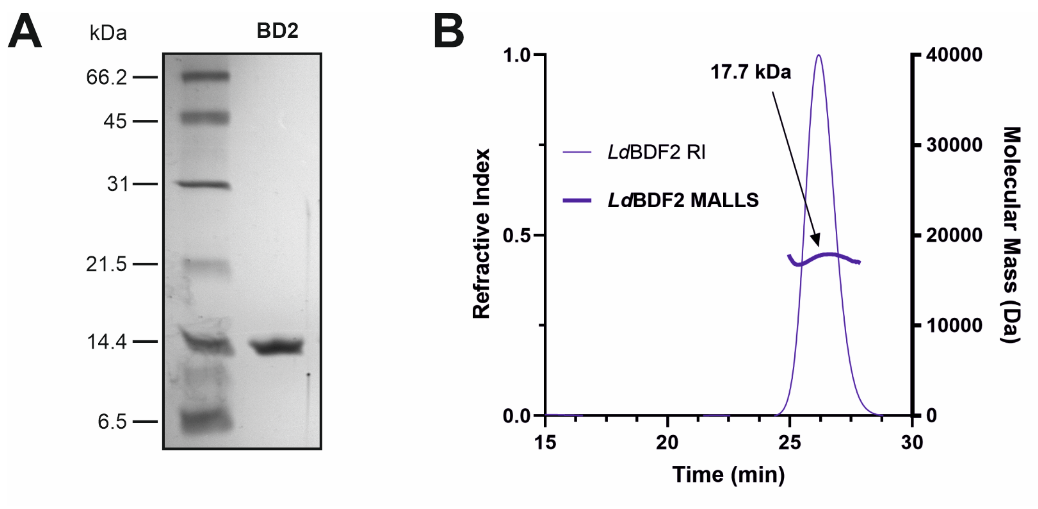

Figure S1. (A) 17.5% SDS-PAGE analysis of purified recombinant His-tag-cleaved BD2 with an expected molecular mass of 14.5 kDa. (B) SEC-MALLS analysis of recombinant His-tagged BD2 with arrow indicating MALLS curve labelled with the associated estimated molecular mass (predicted molecular mass 16.6 kDa).

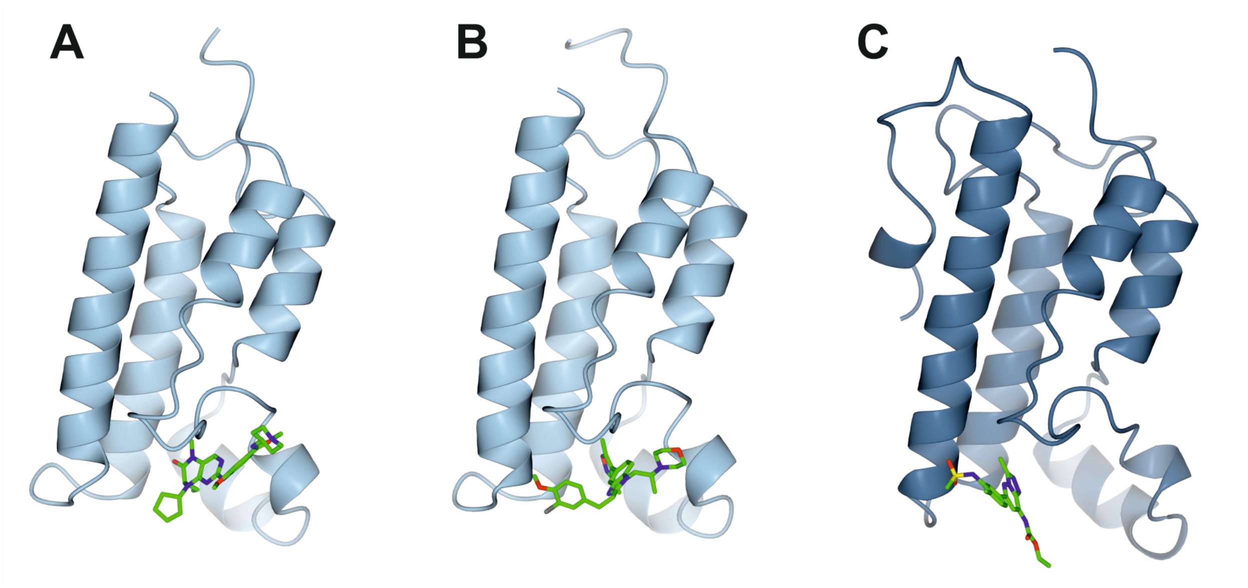

Figure S2. X-ray co-crystal structures of (A) *Ld*BDF BD5.1 in complex with BI 2536, (B) *Ld*BDF BD5.1 in complex with SGC-CBP30, and (C) *Ld*BDF5 BD5.2 in complex with bromosporine. PDB codes are 5TCM, 6BYA & 5TCK, respectively. Figures generated using CCP4mg software.

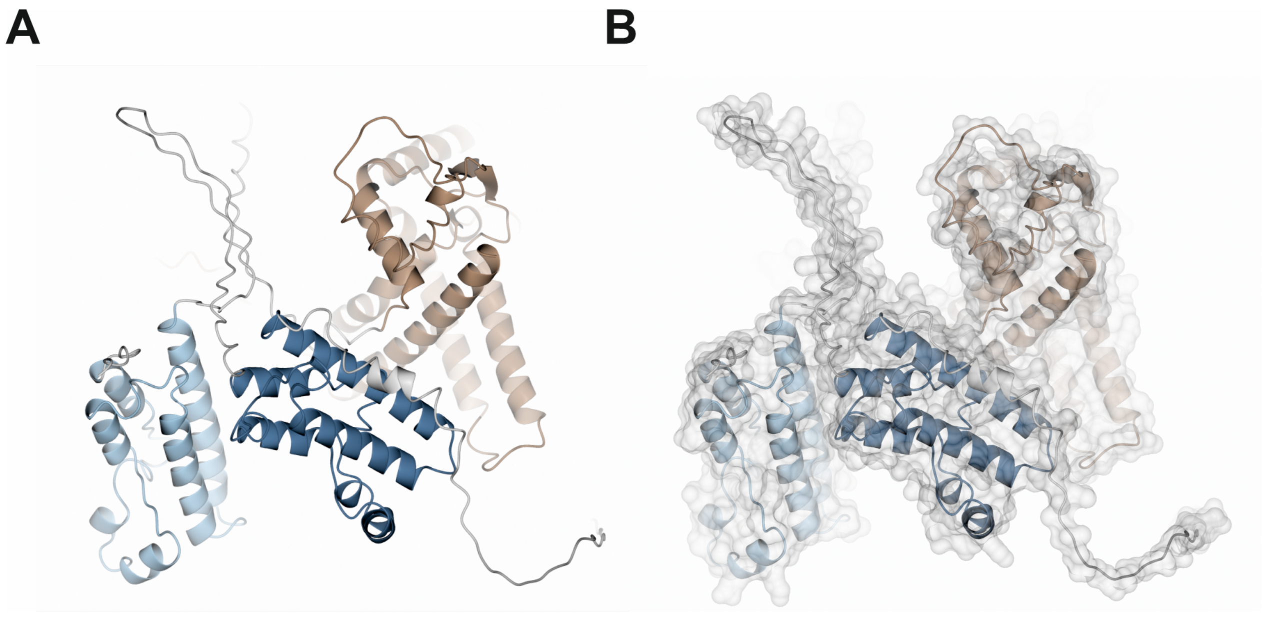

Figure S3. AlphaFold predicted structure of *Ld*BDF5 (LdBPK_091320, AF-A0A3Q8I8I6-F1-model_v4) showing (A) ribbon and (B) surface representations. Different colours indicate the different domains, BD5.1 (light blue), BD5.2 (dark blue) and predicted C-terminal MRG domain (brown).

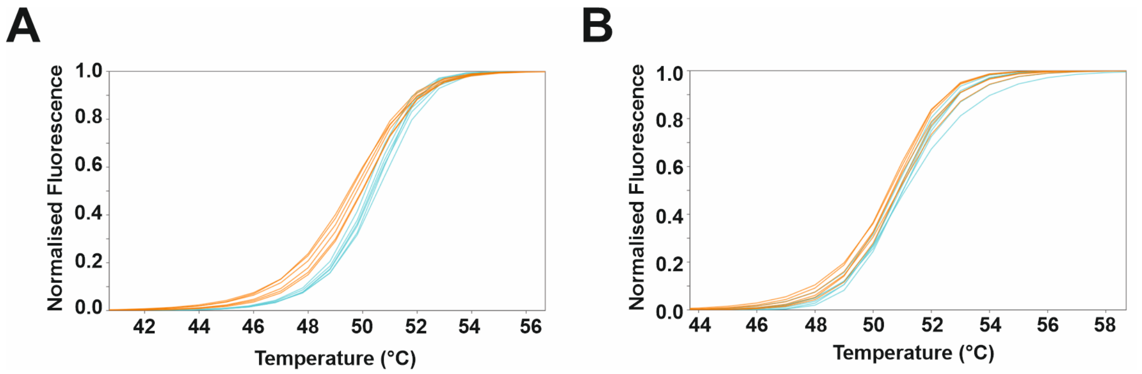

Figure S4. Thermal shift assay melting curves for *Ld*BDF5 BD5T with (A) pan-acetylated $H2B_{9\text{-}23}K9^{Ac}K15^{Ac}K19^{Ac}K21^{Ac}$ and (B) unmodified $H2B_{9\text{-}23}$ peptides at 400 µM. Curves are normalised for five parameter sigmoid equation model fitting for six replicate samples of protein with peptides (blue) alongside DMSO control samples (orange). Graphs produced using the online JTSA tool.

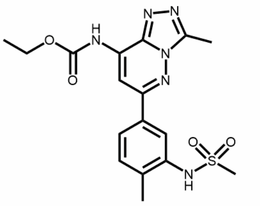

Bromosporine (I-VII)
BD5.1 ΔTm = -1.11 °C (10 μM)
BD5.2 ΔTm = -0.09 °C (10 μM)
BD5T ΔTm = -0.05 °C (25 μM); -0.07 °C (75 μM)

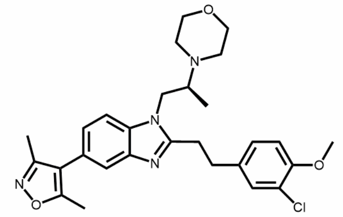

SGC-CBP30 (III)
BD5.1 ΔTm = 1.88 °C (10 μM)
BD5.2 ΔTm = -0.13 °C (10 μM)
BD5T ΔTm = 1.21 °C (25 μM)

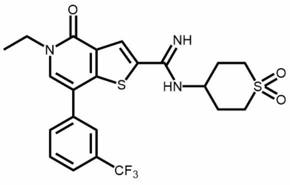

I-BRD9 (IV)
BD5.2 ΔTm = 0.39 °C (25 μM); 0.57 °C (75 μM)
BD5T ΔTm = 0.31 °C (75 μM); 0.81 °C (150 μM)

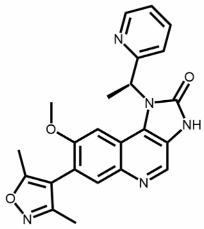

I-BET151 (II)
BD5.1 ΔTm = -0.12 °C (10 μM)
BD5.2 ΔTm = -0.09 °C (10 μM)
BD5T ΔTm = -0.16 °C (25 μM)

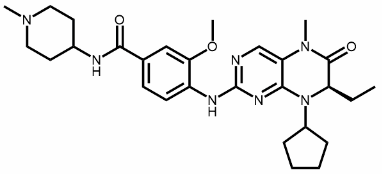

BI 2536 (II)
BD5.1 ΔTm = 0.14 °C (10 μM)
BD5.2 ΔTm = -0.14 °C (10 μM)
BD5T ΔTm = -0.34 °C (25 μM)

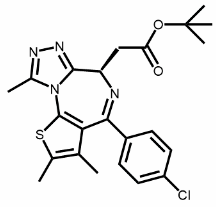

JQ1 (II)
BD5.1 ΔTm = 0.29 °C (10 μM)
BD5.2 ΔTm = -0.05 °C (10 μM)
BD5T ΔTm = -0.57 °C (25 μM)

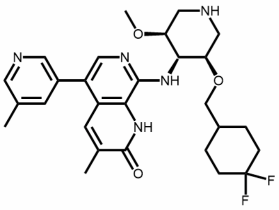

GSK8814 (IV)
BD5.1 ΔTm = -1.14 °C (10 μM)
BD5.2 ΔTm = -0.03 °C (10 μM)

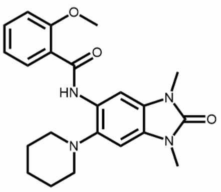

GSK5959 (IV)
BD5.2 ΔTm = -0.18 °C (25 μM)
BD5T ΔTm = -0.60 °C (25 μM)

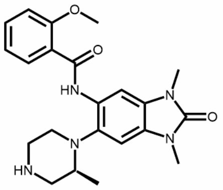

GSK6853 (IV)
BD5.2 ΔTm = 0.06 °C (25 μM)
BD5T ΔTm = -0.17 °C (25 μM)

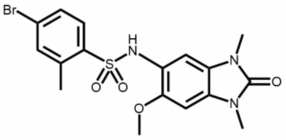

OF-1 (IV)
BD5.2 ΔTm = 0.10 °C (25 μM)
BD5T ΔTm = -0.34 °C (25 μM)

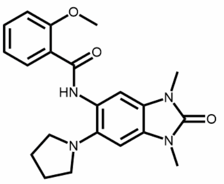

PFI-4 (IV)
BD5.2 ΔTm = -0.08 °C (25 μM)
BD5T ΔTm = -0.23 °C (25 μM)

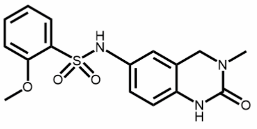

PFI-1 (II)
BD5.2 ΔTm = 0.11 °C (25 μM)
BD5T ΔTm = -0.14 °C (25 μM)

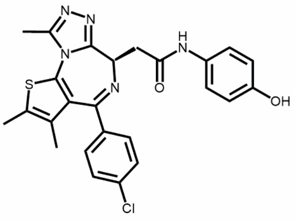

OTX015 (II)
BD5.2 ΔTm = 0.04 °C (25 μM)
BD5T ΔTm = -0.55 °C (25 μM)

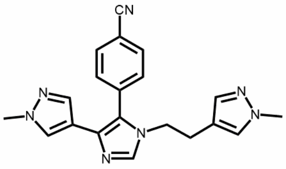

BAZ2-ICR (V)
BD5.2 ΔTm = -0.02 °C (25 μM)
BD5T ΔTm = -0.25 °C (25 μM)

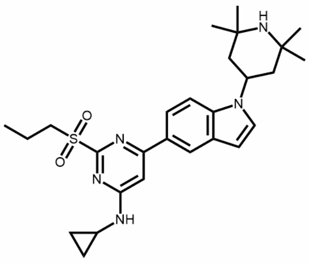

NVS-CRCR2-1 (NVS-1) (I)
BD5.2 ΔTm = 0.21 °C (25 μM)
BD5T ΔTm = -0.01 °C (25 μM)

Figure S5. Structures of 15 human bromodomain inhibitor compounds and the family of human protein target(s) in brackets. Thermal shifts from TSA screen with the *Ld*BDF5 recombinant proteins given below, where compound concentrations are given in brackets.

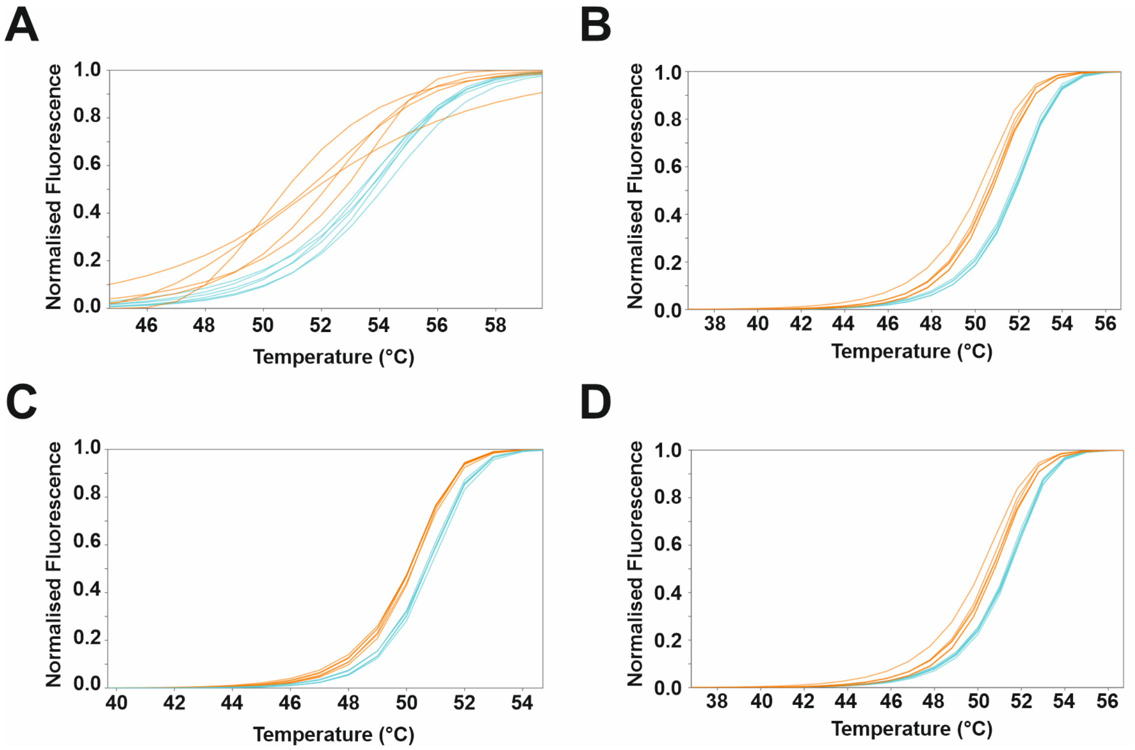

Figure S6. Thermal shift assay melting curves for (A) BD5.1 + SGC-CBP30 at 10 µM, (B) BD5T + SGC-CBP30 at 25 µM, (C) BD5.2 + I-BRD9 at 75 µM, and (D) BD5T + I-BRD9 at 150 µM. Curves are normalised for five parameter sigmoid equation model fitting for five or six replicate samples of protein with compounds (blue) alongside DMSO control samples (orange).

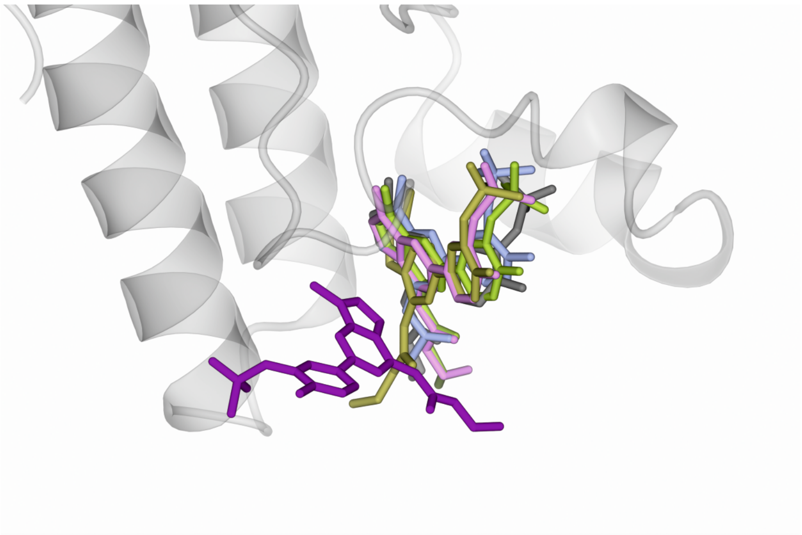

Figure S7. Overlay of co-crystal structures showing bromosporine binding to bromodomains of *Ld*BDF5 BD5.2 (PDB code 5TCK, purple); *Ld*BDF2 (PDB code 5C4Q, blue); *Ld*BDF3 (PDB code 5FEA, yellow), human BRD4 (PDB code 5IGK, grey); human BRD7 (PDB code 6V1H, pink); and human BRD9 (PDB code 5IGM, green). Protein structure of *Ld*BDF5 BD5.2 is shown as ribbon representation.

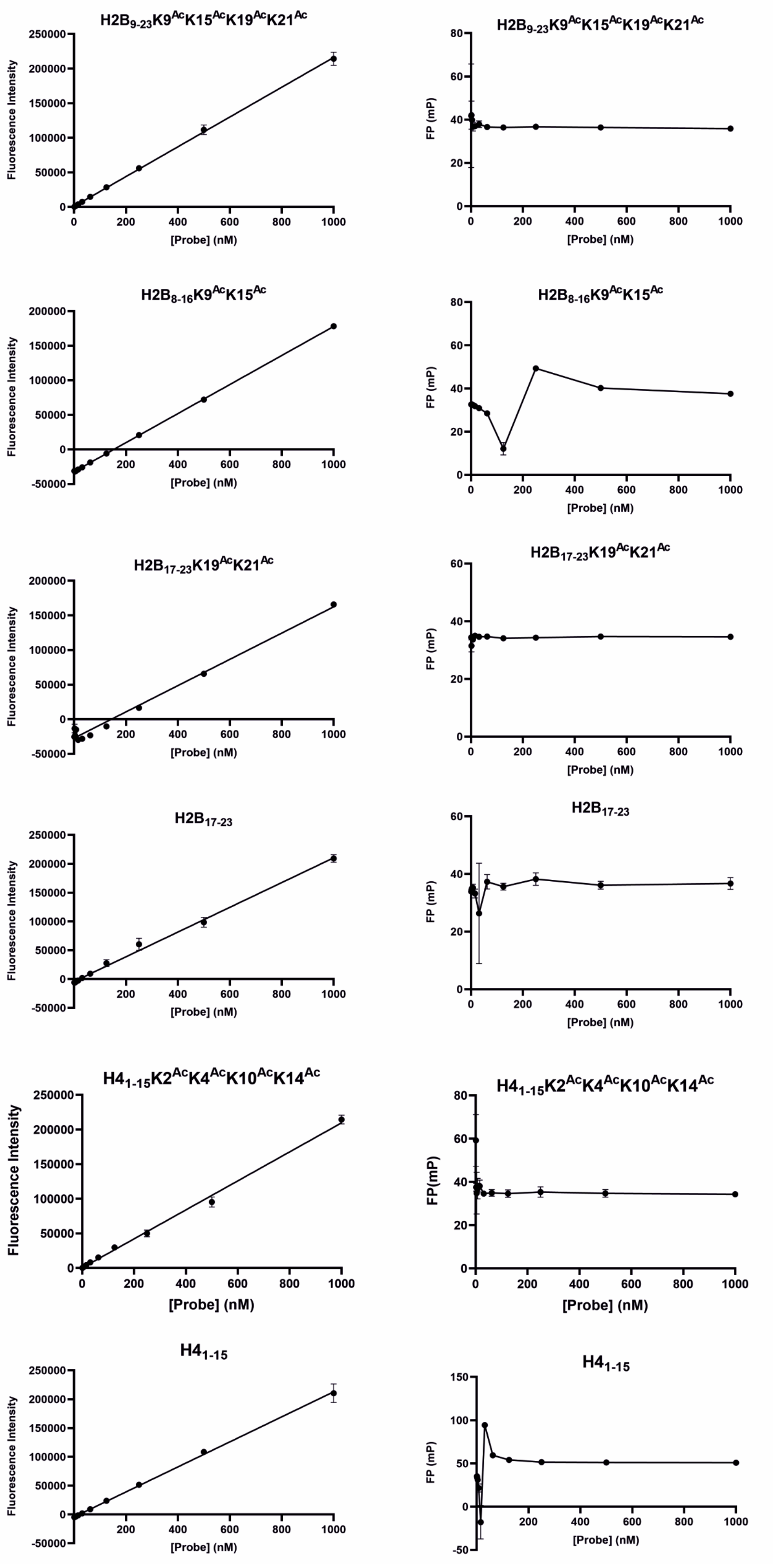

Figure S8. FP probe optimisation with (A) fluorescence intensity and (B) fluorescence polarisation recorded for each peptide probe. Mean, blank-corrected values are plotted against probe concentration and fluorescence intensity data is fitted to linear regression. Error bars represent SD (n = 3).
